# Design and Implementation of Novel Testing System for Intelligent Tire Development: From Bench to Road

**DOI:** 10.3390/s25082430

**Published:** 2025-04-12

**Authors:** Ti Wu, Xiaolong Zhang, Dong Wang, Weigong Zhang, Deng Pan, Liang Tao

**Affiliations:** 1 School of Instrument Science and Engineering, Southeast University, Nanjing 210096, China; wuti@seu.edu.cn; 2 School of Engineering, Anhui Agricultural University, Hefei 230036, China; xlzhang@ahau.edu.cn (X.Z.); 2024094@tlu.edu.cn (L.T.); 3 Giti Tire (China) R&D Center, No. 88 Danxia Road, Hefei 230601, China; pan.deng@giti.com

**Keywords:** intelligent tire technology, accelerometer, PVDF sensor, strain, vertical load estimation, research platform, road testing, wheel force transducer

## Abstract

Intelligent tire technology significantly enhances vehicle performance and driving safety by integrating sensors and electronics within the tire to facilitate the real-time monitoring of tire–road interactions. However, its testing and validation face challenges due to the absence of integrated bench and road testing frameworks. This paper introduces a novel, comprehensive testing system designed to support the full lifecycle development of intelligent tire technologies across both laboratory and real-world driving scenarios, focusing on accelerometer and strain-based sensing. Featuring a modular, distributed architecture, the system integrates an instrumented wheel equipped with multiple embedded tire sensors and a wheel force transducer (WFT), as well as vehicle motion and driving behavior sensors. A robust data acquisition platform based on NI CompactRIO supports multiple-channel high-precision sensing, with sampling rates of up to 50 kHz. The system ensures that data performance aligns with diverse intelligent tire sensing principles, supports a wide range of test parameters, and meets the distinct needs of each development stage. The testing system was applied and validated in a tire vertical load estimation study, which systematically explored and validated estimation methods using multiple accelerometers and PVDF sensors, compared sensor characteristics and estimation performance under different installation positions and sensor types, and culminated in a product-level assessment in road conditions. The experimental results confirmed the higher accuracy of accelerometers in vertical load estimation, validated the developed estimation algorithms and the intelligent tire product, and demonstrated the functionality and performance of the testing system. This work provides a versatile and reliable platform for advancing intelligent tire technologies, supporting both future research and industrial applications.

## 1. Introduction

Intelligent tires, equipped with advanced sensors and real-time communication capabilities, assist in the dynamic monitoring and analysis of tire–road interactions [1]. By providing critical data such as tire forces, road conditions, and vehicle dynamics, they offer valuable solutions in areas like vehicle safety control [2,3,4,5], fuel efficiency [6], autonomous driving [7,8], and road condition assessment [5,9,10,11,12,13].

Various sensing technologies, from basic Tire Pressure Monitoring Systems (TPMSs) [14] to advanced methods like high-speed cameras [15], triboelectric nanogenerators [16], optical fibers [17], and laser-induced graphene sensors [18], have been explored for intelligent tires. Among them, accelerometers [2,3,4,5,12,13,19,20,21,22,23,24] and strain-based sensors, including strain gauges [25,26,27,28,29] and Polyvinylidene Fluoride (PVDF) sensors [11,30,31,32], stand out for their excellent sensing performance and cost-effectiveness, making them research hotspots and key contributors to the recent advancements in intelligent tire development [1].

These sensors, integrated into the tire’s inner liner, actively monitor behaviors like acceleration, deformation, vibrations, and applied forces. Benefiting from recent advancements in data processing technologies, such as machine learning [3,12,19,23,32], effective methods for analyzing sensor signals have been developed, enabling the estimation of key tire parameters, including the tire force [4,19,27], slip angle [2,30,32], tire wear [21,22,23,24], and friction coefficient [11,26,28]. Considering their inherent opacity and limited interpretability, these methods strongly rely on large and comprehensive datasets to ensure robust model training. All relevant factors that may influence the sensor signals and target parameters must be considered during exploration and validation tests, as neglecting any of these factors could lead to unreliable performance in real-world scenarios, even if the algorithms demonstrate strong efficacy under controlled conditions. This underscores the need for comprehensive data acquisition and accurate experimental design.

Despite the growing interest in intelligent tire technologies, real-world applications remain limited due to several gaps. A major challenge is the lack of comprehensive road testing. While most studies rely heavily on bench tests [20,21,23,26,31] or simulations [12,33], these methods fail to capture the complexity of real-world conditions and full-vehicle dynamics. Some studies have attempted road testing, but they often lack comprehensive signal collection for full validation or focus only on simplified, tightly controlled driving scenarios. For example, while [32] conducted road tests and collected tire sensor signals, it did not simultaneously capture other key variables or target parameters, thus failing to validate the road performance of the developed intelligent tire algorithm. Similarly, ref. [24] employed a higher-performance road testing system integrating core tire sensors and a GPS but still lacked critical measurements, relying instead on controlled constants for the tire pressure, vertical load, and vehicle speed. However, real-world conditions cannot be precisely controlled like bench tests; the vehicle speed fluctuates with the driver input, the load distribution shifts with acceleration, and the tire pressure varies with the temperature, factors that should ideally be calibrated through accurate real-time measurements. These limitations stem from the lack of dedicated testing systems that support the synchronized acquisition of both tire sensor data and key test conditions, including dynamic tire forces and vehicle motion.

Beyond the challenges of road testing, another limitation is that most experimental setups are designed for specific research objectives rather than as adaptable platforms for continuous development. When new needs arise, these systems often require redevelopment. This issue is further compounded by the fact that most setups are temporary, often employing separate data acquisition systems that lack real-time synchronization. For instance, in many bench tests [19,25], loading forces and in-tire signals are collected independently, making them unsuitable for dynamic analysis.

Another key limitation is the performance constraints of existing systems. Due to the challenges posed by tire rotation in sensor deployment and signal transmission, many studies adopt cost-effective solutions like wireless transmission and battery-powered setups [29,31,34]. While these approaches simplify deployment, they compromise performance, failing to support high-frequency, multi-channel, or large-volume data acquisition, especially when numerous channels are involved. This limitation restricts certain types of research, such as frequency-domain analysis, which requires high-performance data collection capabilities.

To address the challenges in testing and validating intelligent tires, we developed a novel, comprehensive testing system to support the full lifecycle of intelligent tire technologies. By integrating a modular architecture and advanced sensor technology, the system ensures data performance aligns with diverse intelligent tire sensing principles, supports a wide range of test parameters, and meets the distinct needs of each development stage. Key contributions include the following:

- Full lifecycle testing with dynamic road support;
- Extensive data collection on tire performance, dynamic wheel forces, vehicle motions, and driving behaviors;
- High-performance data acquisition enabled by a modular and distributed architecture.

This paper is organized as follows: Section 2 outlines the design requirements for the proposed system, based on background research into sensing principles, key relevant parameters, and development stage needs. Section 3 details the system architecture, sensor selection, and data acquisition strategies. Section 4 describes how we validated the system’s functionality through bench and road tests, and Section 5 concludes with key contributions and future directions in intelligent tire research.

## 2. Background and Design Requirements

This section establishes the fundamental requirements by analyzing the sensing principles, identifying key measurement parameters, and examining the testing needs at different development stages. First, the mainstream and potential testing methodologies are reviewed to determine the necessary sensor types, sampling frequencies, and channel configurations. Next, critical parameters influencing intelligent tire performance are identified, serving as both key influencing factors and potential research targets. Finally, to facilitate the transition from methodology development to real-world applications, an analysis of scalability and compatibility requirements is conducted to accommodate the varying demands across different development phases.

### 2.1. Sensing Principles and Methodologies for Intelligent Tires

Based on the sensor advantages outlined previously, this study focused on accelerometers and strain-based sensors as the core sensors for intelligent tire systems. Specifically, the Integrated Electronics Piezoelectric (IEPE) triaxial accelerometer and PVDF sensors were selected as representative examples for the subsequent discussions and experimental cases.

**Figure 1:**
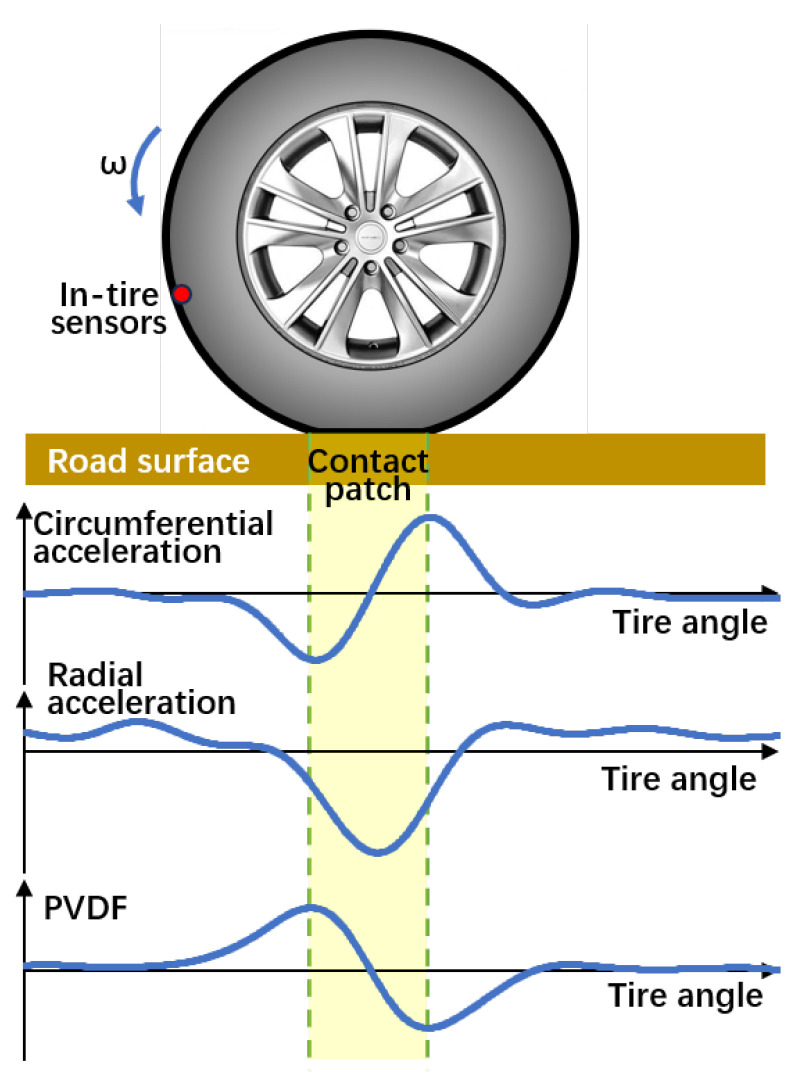
Waveforms of circumferential and radial accelerometer signals and PVDF output during tire ground contact, based on experimental data filtered with a low-pass filter. The yellow area indicates the contact patch.

Sensors are installed on the inner surface of the tire to measure deformations, forces, and vibrations during rotation. Accelerometers detect changes in acceleration due to shifts in the tire’s trajectory, while PVDF sensors capture variations in mechanical stress from ground contact, as shown in Figure 1. These sensors reveal characteristic signal patterns, such as peaks, valleys, and edges at the contact patch. These patterns are then used to calculate the contact length and estimate the vertical load, which form the basis for various measurement strategies in intelligent tire systems [13,19,25,26,29].

In addition to time-domain analysis, the frequency-domain characteristics of sensors [12,13,20] are also critical for accurate tire behavior estimation. High-frequency data acquisition is essential to achieve the resolution needed for such analyses.

Utilizing multi-channel configurations is an effective approach to enhance the sensing ability [11] and achieve an optimal configuration [32], requiring the synchronization of multi-channel signals. Additionally, integrating data from various sensor types enables multi-source data fusion [35,36] and facilitates comparative analyses of different sensing methods to identify their respective advantages and limitations.

In summary, these sensing principles highlight the need for a high-performance testing system capable of supporting high sampling rates, synchronized multi-channel acquisition, and the real-time processing of diverse signal types.

### 2.2. Parameters of Interest in Intelligent Tire Testing

Accurately measuring the parameters that define tire–road interactions and vehicle dynamics is crucial for intelligent tire testing. These parameters not only serve as targets for estimation but also act as experimental variables influencing sensor responses.

In real driving environments, sensor signals are shaped by a range of interacting factors and cross-sensitivities. While existing studies have explored many critical parameters, Table 1 lists commonly studied factors that are not only key research targets but also influence sensor outputs, providing a basic reference for our testing considerations. However, a number of important interactions remain underexamined. For instance, vertical load estimation often combines accelerometer signals with the tire pressure and rotation speed but neglects the subtle effects of lateral forces and movements [21], which can impact accuracy in real-world conditions.

To address these challenges, the test system must account for a comprehensive set of parameters affecting the sensor response. These include tire force information (e.g., vertical and lateral forces), tire–vehicle motion dynamics (e.g., slip and rotation), basic in-tire conditions (e.g., pressure and temperature), and raw sensor signals. These parameters form the foundation for the functional requirements of the testing system.

### 2.3. Development Stages and Corresponding Testing Needs

**Figure 2:**
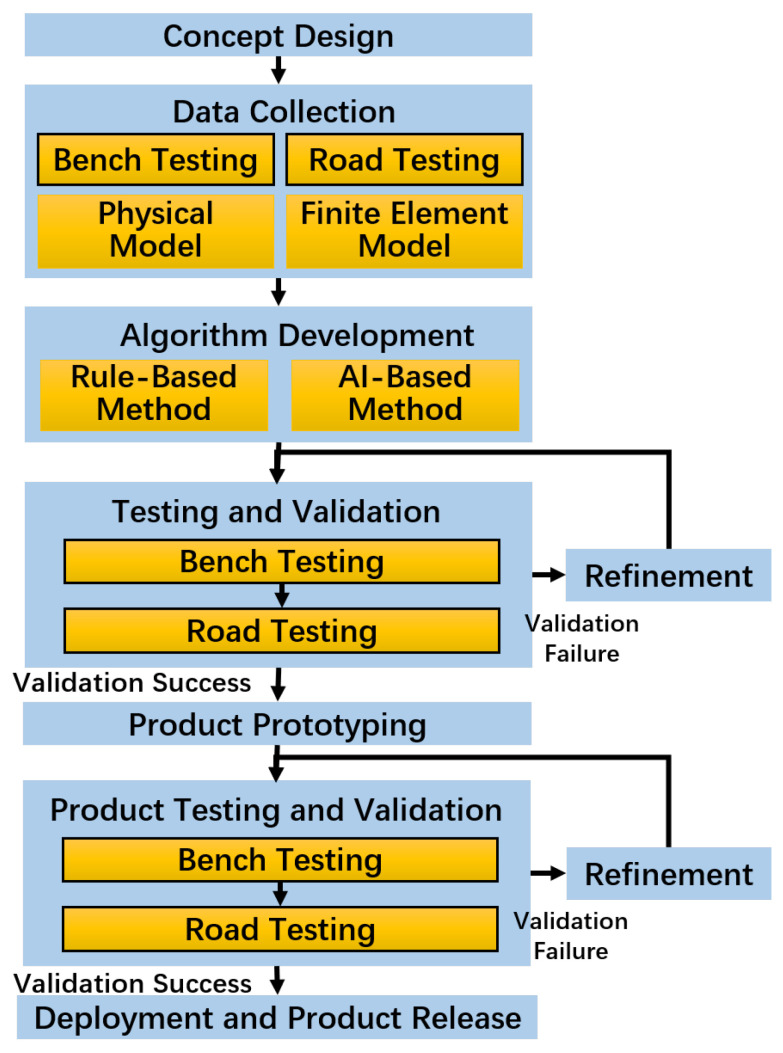
Typical development process for intelligent tire technology.

The development of intelligent tire technology, which currently relies primarily on data-driven approaches, typically spans several phases, as illustrated in Figure 2, each with distinct testing requirements. This phased development cycle demonstrates the importance of a flexible and extensible testing system.

In the early exploration stage, bench testing plays a central role in validating sensing principles and exploring data-driven methods for intelligent tire development. This phase requires the collection of large volumes of diverse and high-quality data to uncover relationships among parameters and to build a sufficient dataset for advanced data processing.

During system-level validation, a combination of bench and road tests is necessary to evaluate sensor layouts and refine data models. This stage requires a testing system that can perform reliably in both controlled laboratory and real-world environments, emphasizing the need for versatility and adaptability in test equipment, particularly the ability to dynamically measure key parameters, including both tire behavior and the vehicle status under various road conditions. However, most existing research has been limited to this stage, focusing primarily on bench testing and lacking sufficient road validation.

In the final product-level assessment, road testing is essential for assessing the performance of the intelligent tire as part of the broader vehicle system. This phase involves calibration, evaluation, and troubleshooting, requiring the testing system to interface with product-specific standards like the Controller Area Network (CAN) bus. Simultaneously, it must support the acquisition of high-performance reference signals to ensure precise calibration and enable detailed defect analysis.

### 2.4. Summary of Design Requirements

Building on the identified sensing principles, key parameters, and testing needs for each development stage, the proposed testing system must fulfill several critical requirements to support intelligent tire development throughout all stages:

- **The Complete Integration of Tire Measurement Parameters:** This ensures the comprehensive measurement of tire-related information, including tire force information (e.g., vertical and lateral forces), tire–vehicle motion dynamics (e.g., speed, cornering, slip), basic in-tire conditions (e.g., pressure and temperature), and raw sensor signals (e.g., accelerometers, strain gauges, PVDF sensors). This guarantees that all relevant factors affecting tire performance are captured and considered during the testing process.
- **High-Frequency Multi-Channel Sampling for Core Tire Sensors with Cross-Sensor Synchronization:** The sampling frequency of the core tire sensors (typically recommended to be no less than 2 kHz, with this study adopting 50 kHz) must meet the requirements for frequency-domain analysis and capture critical sensor characteristics by providing sufficient samples per tire rotation, even at high rotation speeds (e.g., at 120 km/h, a 2 kHz sampling rate provides 116 samples per rotation for a 205/55 R16 tire). Furthermore, synchronization with other sensors operating at varying frequencies is essential. For example, in-tire accelerometers and PVDF sensors typically operate in the kilohertz range, vehicle motion sensors in the tens of Hz, and tire pressure and temperature sensors at much lower frequencies, typically below 1 Hz. Adopting a hardware-level synchronization mechanism, such as an FPGA (Field-Programmable Gate Array), is critical to ensure their accurate integration.
- **Modular and Flexible Design for Functional Expansion and Seamless Integration with Vehicle Systems:** The system should be easily reconfigurable for various testing scenarios (e.g., bench tests, road validation, product testing) and include reserved interfaces (e.g., a standardized CAN bus) for seamless integration with additional sensors and vehicle systems.
- **Efficient Data Processing and Real-Time Performance Achieved Using a Distributed Architecture:** This facilitates the efficient processing of large datasets, such as those required for machine learning, while ensuring real-time performance across sensors. A distributed architecture is essential to effectively allocate tasks, providing greater computational power and storage efficiency at higher processing levels while maintaining real-time performance at the sensor interface and data acquisition levels.
- **Robustness and Durability:** These ensure reliable performance in harsh testing environments, including when high temperatures, vibrations, and mechanical stresses are experienced within the tire.

## 3. System Design

### 3.1. Overall System Architecture

**Figure 3:**
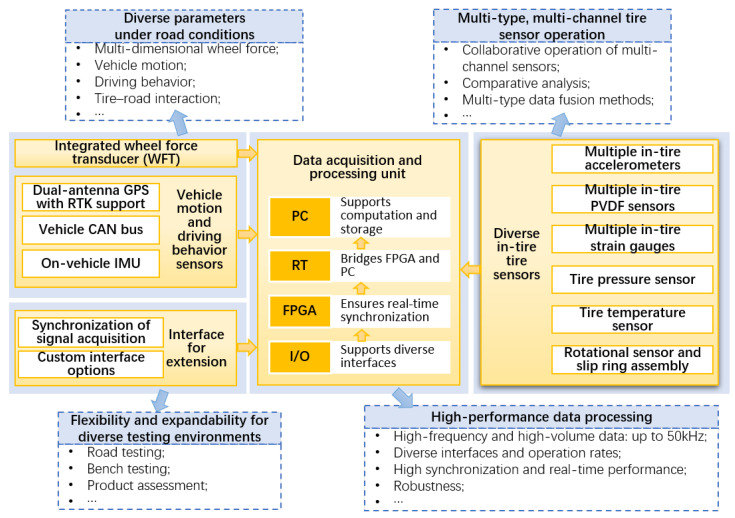
Overall design of the testing system, including strategies to address system challenges and the modular architecture.

**Figure 4:**
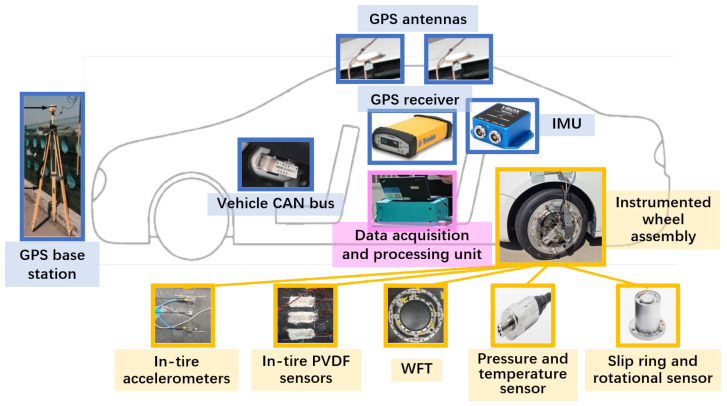
A road implementation of the overall testing system design. The yellow arrow highlights the instrumented wheel assembly and its key sensing components.

To meet the diverse requirements of intelligent tire development, the proposed system adopts a modular and distributed architecture. The overall design of the testing system, including strategies to address system challenges, is illustrated in Figure 3. A road implementation that concretely demonstrates the testing system design is shown in Figure 4. At the core of this design, the NI CompactRIO-based data acquisition and processing unit ensures the seamless integration of key system components, including the instrumented wheel with embedded tire sensors and a WFT, vehicle motion and driver behavior sensors, and standardized interfaces, collectively supporting both bench testing and real-world scalability.

### 3.2. Instrumented Wheel Assembly

The instrumented wheel, equipped with a multi-dimensional WFT and multiple in-tire sensors, acquires key tire data and serves as the core component of the sensor system.

- **Intelligent Tire Sensors:** Multiple IEPE triaxial accelerometers and PVDF sensors are strategically mounted on the tire’s inner liner to capture their response characteristics under varying operating conditions. Their placement supports the investigation of positional and orientational effects, as well as the interrelationships between different sensing modalities.
- **Tire Pressure and Temperature Sensor:** To ensure higher accuracy and greater real-time monitoring compared to a conventional TPMS, a combined thin-film pressure and temperature sensor (PCM167, EFE Sensors, Goleta, CA, USA), integrating a Resistance Temperature Detector (RTD) Pt-1000 element, is incorporated into the modified wheel rim to measure the in-tire pressure and temperature.
- **Slip Ring with Rotational Sensor:** To manage the substantial data generated by these sensors, a slip ring is employed to maintain a wired connection, ensuring superior reliability, bandwidth, and real-time data transmission in harsh working environments. Additionally, the slip ring is integrated with a rotational sensor that provides wheel rotation angle data via quadrature-encoded pulses. These data are crucial for the accurate determination of the wheel speed and angular position of the in-tire sensors, enabling precise synchronization with other system components.

### 3.3. Integration with WFT System

**Figure 5:**
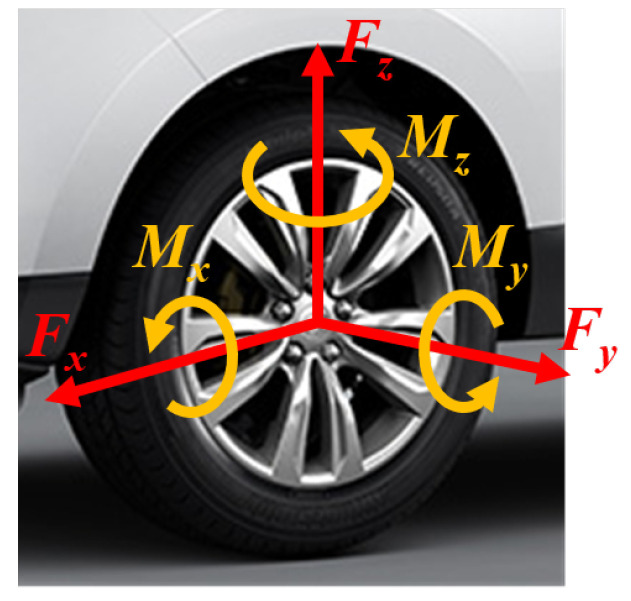
The definition of 6-dimensional wheel forces.

One of the most critical and challenging aspects of intelligent tire testing is the dynamic measurement of multi-dimensional wheel forces, including the longitudinal force $F_{x}$, lateral force $F_{y}$, vertical force $F_{z}$, heeling moment $M_{x}$, twist torque $M_{y}$, and aligning torque $M_{z}$ (see Figure 5), as well as force-related parameters like the tire–road friction coefficient μ [11]:

(1) $$\mu =\frac{F_{x}}{F_{z}}=\frac{\mathrm{Friction}\;\mathrm{force}}{\mathrm{Normal}\;\mathrm{force}}.$$

This can be achieved using WFT systems, such as our previously developed strain-based WFT featuring an eight-beam elastic body [37,38,39,40,41] or other commercial WFT products [42].

To ensure hardware compatibility and real-time performance, modifications to the WFT system are necessary. This paper provides basic sensing principles and data processing methods to illustrate the approach for WFT modification and integration. However, WFT design is a complex task, and the focus of this study was not on its design but on how to integrate it into the testing system to achieve an optimized overall performance. The mechanical implementation of a WFT presented in this paper is a simplified example, and the data processing approach is based on an idealized model without advanced error compensation or nonlinear corrections. The purpose is to provide readers with a conceptual understanding of tire force measurement within the system. For more in-depth discussions on WFT design and force data computation, please refer to our previous studies [37,38,39,40,41].

**Figure 6:**
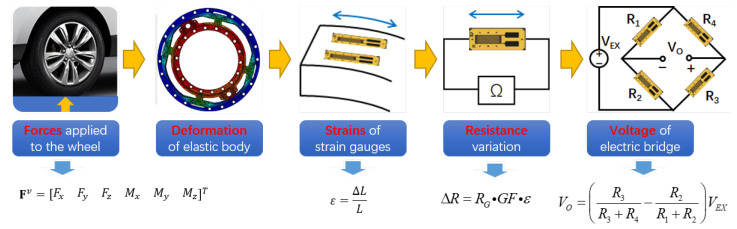
Signal transformation process for sensing the wheel force. $F^{v}$ denotes the six-dimensional wheel force vector in the vehicle coordinate system. The applied forces cause deformation of the elastic body, resulting in strain ϵ in the strain gauges. This strain leads to a resistance change ΔR, which is then converted into a voltage output $V_{O}$ by the electric bridge. Red text highlights key physical quantities; yellow arrows indicate the signal transformation flow, and blue arrows denote the corresponding formula expressions.

**Figure 7:**
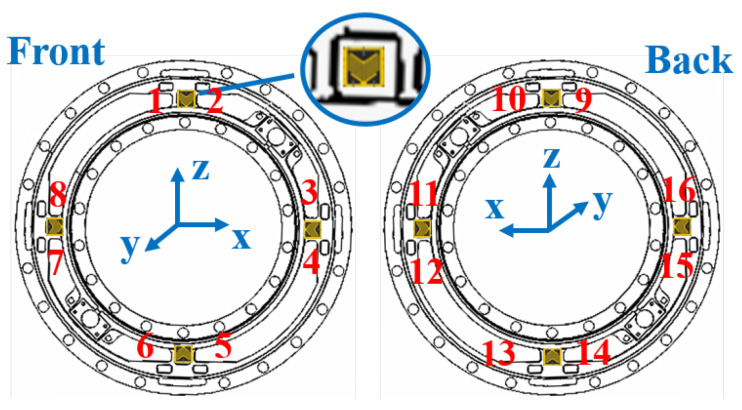
Strain gauge layout on the elastic body. Strain gauges are symmetrically affixed to each of the four beams. The numbered labels correspond to the resistor identifiers in Figure 8, where each bi-axial gauge is represented as two separate elements for clarity.

**Figure 8:**
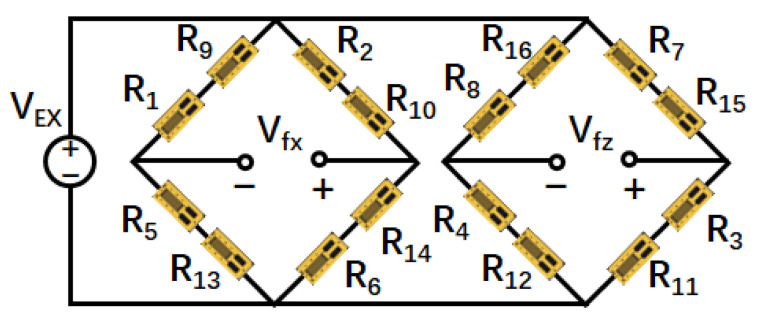
Bridge circuit configuration corresponding to the strain gauges. Bi-axial gauges are illustrated as two distinct elements, matching the strain gauge labels in Figure 7.

**The Sensing Principle of a WFT:** A WFT measures tire forces by converting mechanical forces into electrical signals, as illustrated in Figure 6. Figure 7 and Figure 8 present a mechanical design example of a two-dimensional self-decoupling WFT for measuring $F_{x}$ and $F_{z}$, with a simplified strain gauge layout based on [42]. A specially designed elastic body (Figure 7) integrated into the wheel rim deforms under applied forces, causing resistance changes in the attached strain gauges. These changes are processed using Wheatstone bridges (Figure 8), generating voltage outputs, V, proportional to the applied forces, F:

(2) V=C·F,

where C is the transformation matrix and should be non-singular to ensure unique solutions.

**WFT Signal Processing:** In a self-decoupling WFT, where C is diagonal, force components directly correspond to voltage outputs and can be calculated as

(3) $$f_{i}=\frac{1}{c_{ii}}v_{i},\;i=1,\ldots ,6,\;\left(c_{ij}=0,i\neq j\right),$$

where $c_{ii}$ is typically obtained through a calibration test in a practical implementation.

The calculated force F is initially expressed in the wheel coordinate system and must be transformed into the vehicle coordinate system, denoted as $F^{v}={\left[F_{x}\;F_{y}\;F_{z}\;M_{x}\;M_{y}\;M_{z}\right]}^{T}$, using a transformation matrix, T:

(4) $$F^{v}=T\cdot F,$$

(5) $$T=\left[\begin{array}{cccccc} \cos\theta & 0 & \sin\theta & 0 & 0 & 0\\ 0 & 1 & 0 & 0 & 0 & 0\\ -\sin\theta & 0 & \cos\theta & 0 & 0 & 0\\ 0 & 0 & 0 & \cos\theta & 0 & \sin\theta \\ 0 & 0 & 0 & 0 & 1 & 0\\ 0 & 0 & 0 & -\sin\theta & 0 & \cos\theta \end{array}\right],$$

where θ, the wheel’s rotation angle, is measured by the rotational sensor and determines the matrix T.

**Integration and Hardware Implementation:** The testing system interfaces directly with the WFT’s electrical bridge circuits, bypassing its signal acquisition and data processing components and instead implementing these functions within its own architecture. This modification eliminates delays caused by pre-processed WFT outputs and ensures synchronized data acquisition.

**Figure 9:**
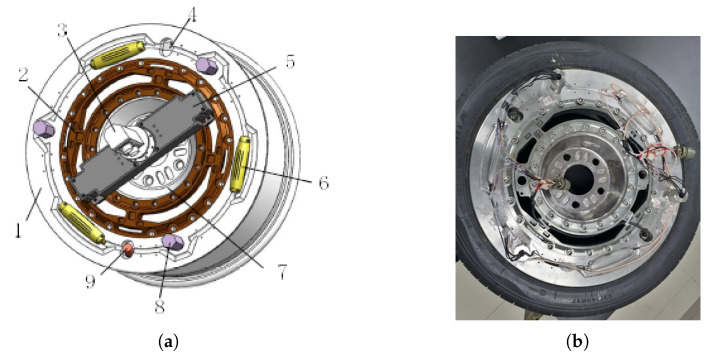
The modified rim with the WFT accessories: (**a**) 3D assembly diagram, (**b**) a picture of the wheel with the elastic body incorporated into the rim. The key components are labeled as follows: 1—the modified rim; 2—the elastic body; 3—the slip ring; 4—the valve core; 5—the box for the electronic board; 6—the charge amplifier and the container for the PVDF sensor; 7—the hub adapter; 8—the hole seal; and 9—the tire pressure and temperature sensor.

Additionally, as shown in Figure 9, the wheel rim needs to be modified to accommodate the WFT’s elastic body, its accessories, and the in-tire sensors, supporting the implementation of the instrumented wheel as demonstrated in our previous studies [43,44].

### 3.4. Vehicle Motion and Driving Behavior Sensing

Vehicle motion and driving behavior are monitored through the combined sensing of the Global Positioning System (GPS), Inertial Measurement Unit (IMU), and the vehicle’s CAN bus.

The GPS provides fundamental kinematic information, including the speed, trajectory, and heading. A dual-antenna GPS with Real-Time Kinematic (RTK) support is recommended to enhance the heading accuracy and provide centimeter-level positioning, enabling precise trajectory reconstruction and the calculation of tire-related parameters, such as the tire slip angle. The IMU complements the GPS by measuring high-frequency dynamic characteristics, including the acceleration, angular velocity, and 3D attitude (pitch, roll, and yaw), which are critical for analyzing the load transfer to tires during braking, cornering, or sudden maneuvers. Vehicle CAN data integrate driver inputs, such as the throttle position, braking force, steering angle, and wheel speed, forming the link between driving behavior and tire performance.

**Figure 10:**
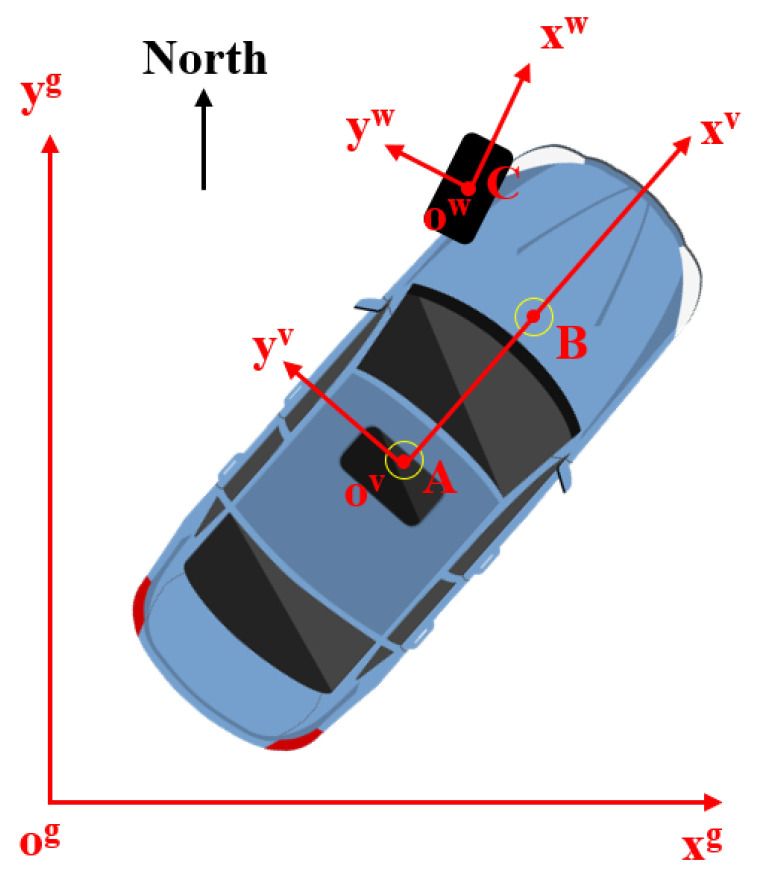
Top view of vehicle motion and 2D planar coordinate system with three coordinate systems: ground (g), vehicle (v), and wheel (w).

It should be noted that our test system incorporates multiple types of sensors, each fixed to distinct coordinate systems, such as the ground (e.g., GPS), vehicle (e.g., IMU), and wheel (e.g., in-tire accelerometers) coordinate systems, as illustrated in the 2D top view in Figure 10. Coordinate transformations may be required when combining information from these sensors.

This configuration provides a foundational framework for measuring driving information in intelligent tire road testing. It can be flexibly expanded or simplified to meet specific experimental objectives. For instance, the following experimental chapter describes how a simplified test setup based on this design was employed to verify the vertical load estimation under varying driving conditions. Additionally, the configuration can determine the slip ratio [3], or, when integrated with a wheel steering angle sensor, calculate the tire slip angle [45], thereby facilitating road testing for estimating either the slip ratio [3] or the tire slip angle [30].

### 3.5. Data Acquisition and Processing Unit

The data acquisition and processing unit is built on the NI CompactRIO platform, enabling high-frequency synchronized sampling at 50 kHz across up to 20 channels. This includes sensors such as accelerometers, PVDF sensors, strain gauges (for the WFT or extensible intelligent tire sensors), and rotational angle sensors. Additionally, lower-frequency signals, such as the 100 Hz tire pressure, temperature, and CAN data and 10 Hz GPS information, are seamlessly integrated and synchronized.

**Figure 11:**
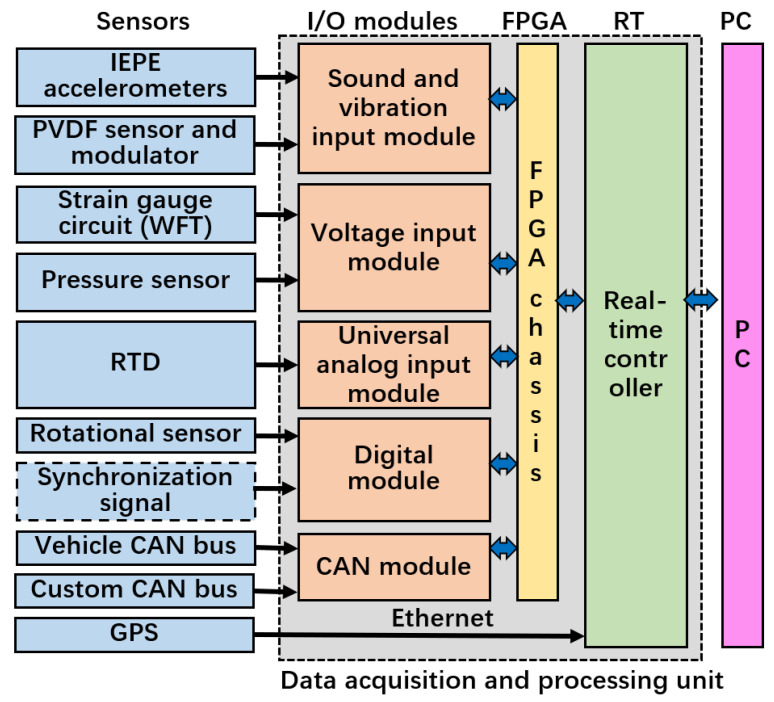
Hardware architecture of the data acquisition and processing unit.

**Figure 12:**
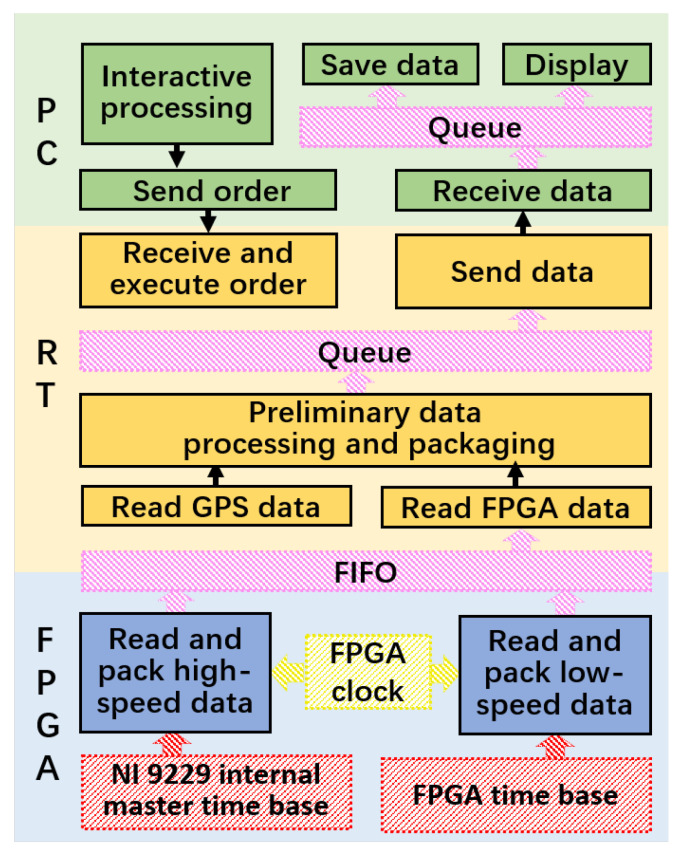
Software architecture of the test system.

The system utilizes a modular and distributed architecture, as illustrated in Figure 11. The software, implemented in LabVIEW 2019, is correspondingly distributed across the FPGA, RT (real-time controller), and host PC (Personal Computer) layers, as shown in Figure 12. By strategically allocating tasks, it ensures real-time performance and synchronization (primarily managed by the FPGA and RT layers), while providing the necessary computational power and storage efficiency (mainly handled by the host PC layer).

- **FPGA layer**: The FPGA is responsible for real-time data acquisition and synchronization. Each sensor type operates in an independent while loop, leveraging the FPGA’s parallel processing capabilities to ensure reliable and efficient performance. High-frequency data acquisition is synchronized using the NI 9229 module’s internal master time base. All data streams are timestamped at the FPGA level, enabling alignment during post-processing.
- **RT layer**: The RT layer bridges the FPGA and the host PC, handling preliminary data processing and data transfer. Communication between the FPGA and RT is facilitated by FIFOs, while queues ensure reliable and lossless transmission to the host PC. The RT also supports GPS signal acquisition in an NMEA format via Ethernet, utilizing the Ethernet interface integrated into the RT controller hardware.
- **Host PC**: The host PC implements a state machine to manage user interactions, advanced data analysis, and logging. Data are stored in a Technical Data Management Streaming (TDMS) format, allowing for efficient organization, metadata tagging, and streamlined access for analysis.
- **Synchronization and task parallelization**: Synchronization across all layers is achieved using FPGA-assigned timestamps, ensuring the precise alignment of high-frequency and low-frequency signals. Each major task—whether data acquisition, pre-processing, or logging—is executed in an independent while loop, enhancing reliability, real-time performance, and logical clarity.

## 4. Bench Test

To demonstrate the effectiveness of the proposed testing system, a comprehensive experimental study was conducted, using the development of a tire vertical load estimation method with in-tire accelerometers and PVDF sensors as an example. This study encompassed key development stages, including bench testing, road validation, and product-level assessment. Since the primary objective was to evaluate the testing system’s data acquisition capabilities and functionality, the focus was placed on presenting raw measurement data rather than conducting an in-depth analysis of specific estimation techniques. A more detailed analysis of these datasets, with a focus on vertical load estimation techniques, can be found in our recent work [46], where the same dataset was employed.

This chapter presents the bench test, focusing on the comparative analysis of signal characteristics and prediction performance for different sensor types, including accelerometers and PVDF sensors.

### 4.1. Experimental Setup

**Figure 13:**
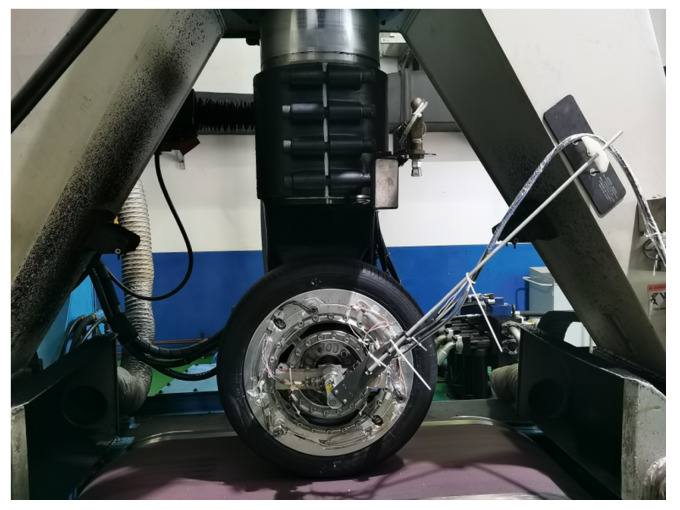
Bench test using MTS Flat-Trac system.

**Figure 14:**
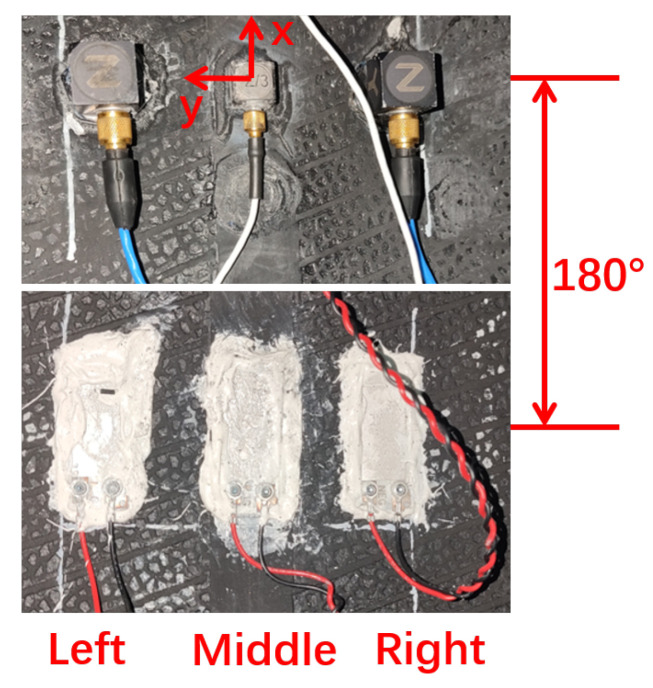
Three accelerometers (**above**) and three PVDF sensors (**below**) mounted on the inner tire liner.

The test was conducted using a radial tire (Giti 225/45 R17) on the MTS Flat-Trac system (MTS Systems Corporation, Eden Prairie, MN, USA), as shown in Figure 13. Three triaxial accelerometers (Model 3333M2T, Dytran Instruments, Inc., Los Angeles, CA, USA) and three PVDF sensors were mounted on the inner tire liner, positioned 180 degrees apart in the circumferential direction (Figure 14). The proposed testing system provided high-frequency sampling at 50 kHz for the three in-tire accelerometers, three PVDF sensors, and rotation angle signals. A lower sampling frequency of 100 Hz was used for the integrated in-tire pressure and temperature measurements. The MTS Flat-Trac system provided a controlled environment and recorded reference data, including the rotation speed and wheel forces.

The experiment controlled the vertical load, tire pressure, and vehicle speed.

### 4.2. Data Acquisition and Integrity

**Figure 15:**
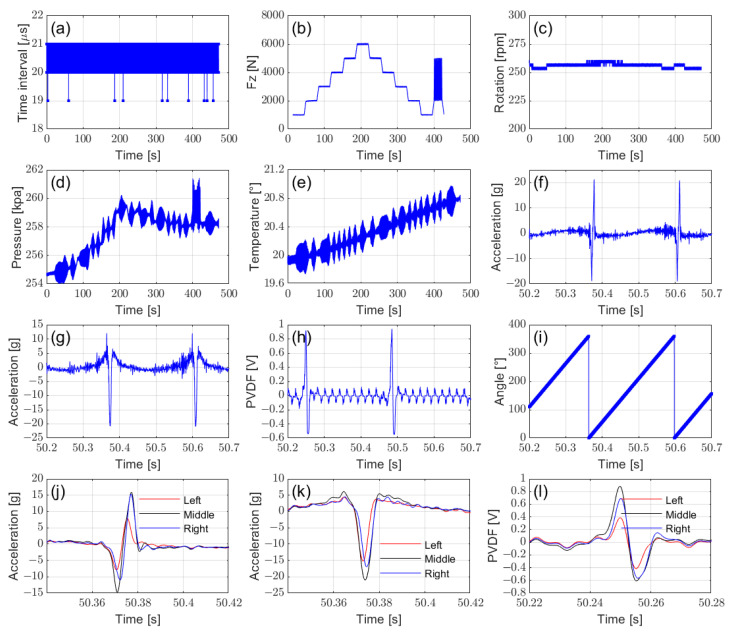
Bench test data curves: (**a**) time interval, (**b**) vertical loading, (**c**) rotation speed, (**d**) tire pressure, (**e**) tire temperature, (**f**) *x*-axis acceleration recorded by middle sensor, (**g**) *z*-axis acceleration recorded by middle sensor, (**h**) PVDF signals recorded by middle sensor, (**i**) tire rotation angle, (**j**) *x*-axis acceleration at different installation positions, (**k**) *z*-axis acceleration at different installation positions, (**l**) PVDF signal at different installation positions.

Figure 15 illustrates the data acquisition details under test conditions with a speed of 30 km/h and a tire pressure of 260 kPa.

Figure 15a presents the time difference curve of the recorded high-frequency data, confirming data integrity with a consistent sampling interval of 20 ms. Handling multiple channels at a 50 kHz sampling rate is challenging; however, the system successfully achieved real-time data acquisition without any data loss.

Figure 15b shows the vertical load procedure, which consisted of a steady-state step-change loading phase followed by a dynamic sinusoidal loading phase. This curve is presented to illustrate that the system supports both steady-state and dynamic testing. Figure 15d,e illustrate the variations in the tire pressure and temperature during the test, emphasizing the necessity of the real-time monitoring of these parameters rather than relying on constant pre-test values in intelligent tire testing.

Figure 15f–h display the signals captured by the middle accelerometer and PVDF sensor through high-frequency sampling, with the corresponding angular position shown in Figure 15i. These signals can be used for a comparative study of different sensor types. The *y*-axis acceleration showed minimal variation and is therefore omitted here. Figure 15j–l compare the characteristics of the same sensor type at different installation positions. This analysis can help assess the impact of sensor placement on signal characteristics and prediction algorithms.

### 4.3. Vertical Load Estimation Analysis

The comprehensive and varied test data support a range of intelligent tire technology research, including the identification of tire contact patch characteristics and the development of vertical load estimation methods. Furthermore, these data enable in-depth performance analysis across various influencing factors, such as sensor types and installation positions.

**Figure 16:**
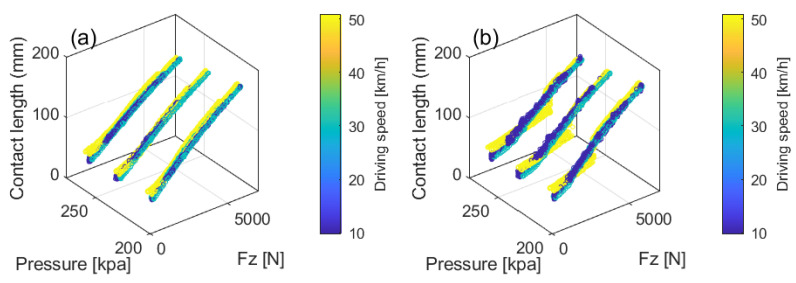
Relationship between contact length, vertical load $F_{z}$, tire pressure, and driving speed: (**a**) contact length calculated based on *x*-axis acceleration; (**b**) contact length calculated based on PVDF sensor data.

Here, we present a research case of a comparative analysis for vertical load estimation based on different sensor types, including accelerometers and PVDF sensors. Figure 16 presents comprehensive testing data collected from the proposed test system with testing variables of vertical loads, speeds, and tire pressures, revealing the factors influencing the contact patch length. The contact patch length was derived from the peak–valley features of *x*-axis acceleration and PVDF sensor data shown in Figure 15f and Figure 15h, respectively, as described by the following equation:

(6) $$L=2R\sin\left(\frac{\pi \theta }{90}\right),$$

where *R* is the rolling radius, and θ is the angular difference between the peak and valley.

**Figure 17:**
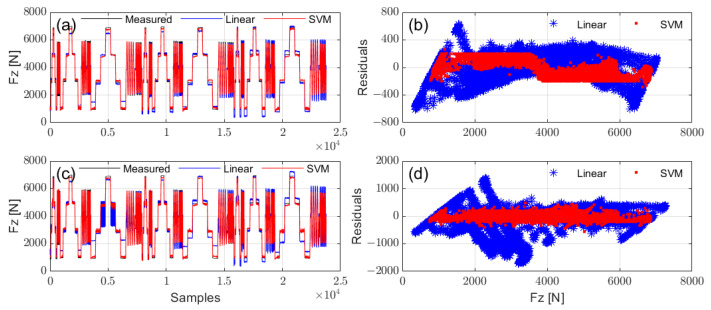
Vertical load estimation results and residuals obtained using different methods: (**a**) estimation from *x*-axis acceleration; (**b**) residuals from *x*-axis acceleration; (**c**) estimation from PVDF signal; (**d**) residuals from PVDF signal.

Then, vertical load estimation was performed using the contact patch length, speed, and tire pressure from Figure 16 as input variables, with both Support Vector Machine (SVM) and linear regression methods applied. The SVM was implemented using a radial basis function (RBF) kernel, with automatic kernel scaling and standardized data. The results are shown in Figure 17, with the corresponding Mean Absolute Percentage Error (MAPE) provided in Table 2.

This was a preliminary test focused on data presentation rather than an in-depth technical analysis. However, simple observations revealed that the PVDF-based contact length estimation exhibited significant fluctuations, particularly at high speeds (Figure 16). Future studies should aim to investigate the causes of these fluctuations with a more detailed analysis.

Regarding the vertical load prediction performance, the accelerometer-based method clearly demonstrated higher accuracy, while the PVDF-based method showed poorer performance, likely due to instability in contact length estimation (Figure 16). Additionally, nonlinear prediction (SVM) significantly outperformed linear regression, which could somewhat mitigate the mechanical limitations of the PVDF sensor.

Although these prediction results indicate the modest accuracy of the vertical load estimation approach, which depends on contact length estimation, this method serves as the foundation for many tire parameter sensing strategies. It is essential to leverage the high-quality raw sensor data provided by the proposed system to analyze the root causes of performance deficiencies, in addition to merely focusing on developing advanced prediction algorithms.

### 4.4. Summary of Bench Test Results

Throughout the tests, the proposed system provided multi-channel, high-frequency, real-time data acquisition for various tire sensors. The core tire sensors reliably operated at a 50 kHz sampling rate without data loss. This capability ensures robust support for intelligent tire technology development, including prediction algorithm development and sensor performance comparisons.

## 5. Road Test

Following the bench tests, the road test validated the vertical load estimation method and provided a product-level assessment for our self-designed Intelligent Tire Test Unit (ITTU) in real-world scenarios. The vertical load predictions were based on data from accelerometers, which performed better in the bench tests.

### 5.1. Experimental Setup and Hardware Architecture

**Figure 18:**
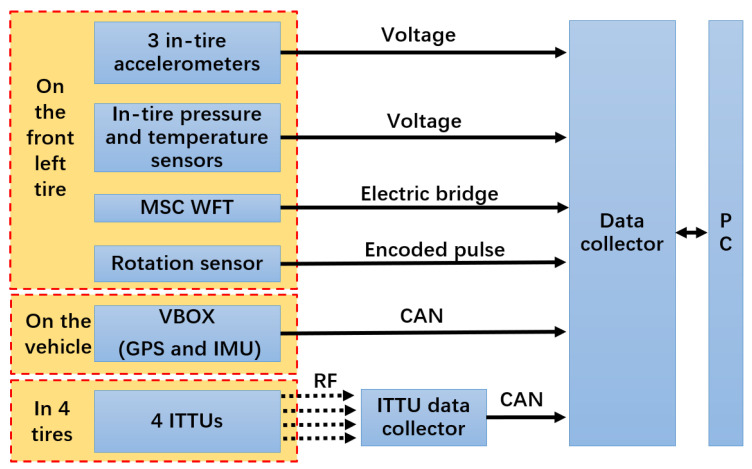
Experimental setup and hardware architecture for the road test. Solid arrows represent wired connections, and dashed arrows indicate wireless links.

**Figure 19:**
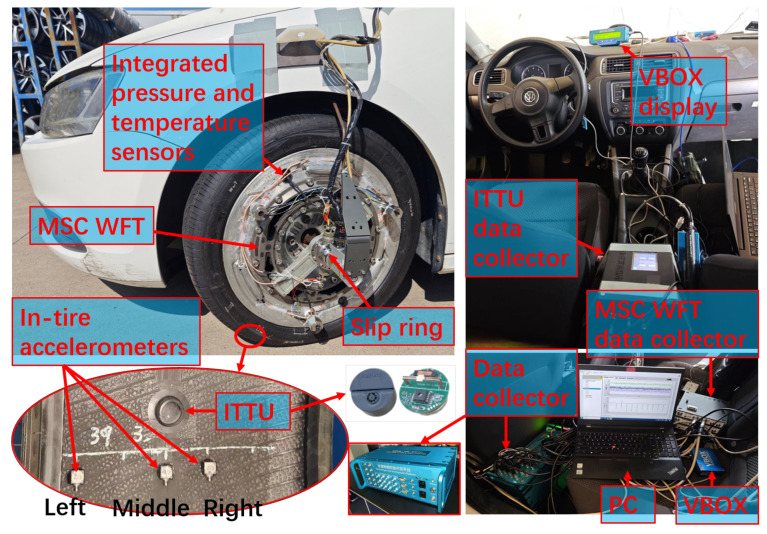
Road test images.

Figure 18 illustrates the test setup and hardware architecture, while Figure 19 depicts the test environment. Three three-axis accelerometers, including one at the center, were asymmetrically mounted on the tire. The WFT system (Michigan Scientific Corporation, Charlevoix, MI, USA) measured 6-dimensional wheel forces and moments for calibration through direct integration into its bridge circuit, while the Racelogic VBOX 3i (Racelogic Ltd., Buckingham, UK) recorded vehicle motion data via the testing system’s custom CAN interface, enabling a detailed analysis of driving conditions.

The ITTU integrates an accelerometer and pressure and temperature sensors into a compact unit, transmitting data via RF to a central collector. The collector consolidates the data and forwards them through the CAN bus. In this test, four ITTUs were mounted on the inner liner of each tire, with the central collector connected to the road validation system via the CAN bus.

### 5.2. Data Acquisition and Analysis

**Figure 20:**
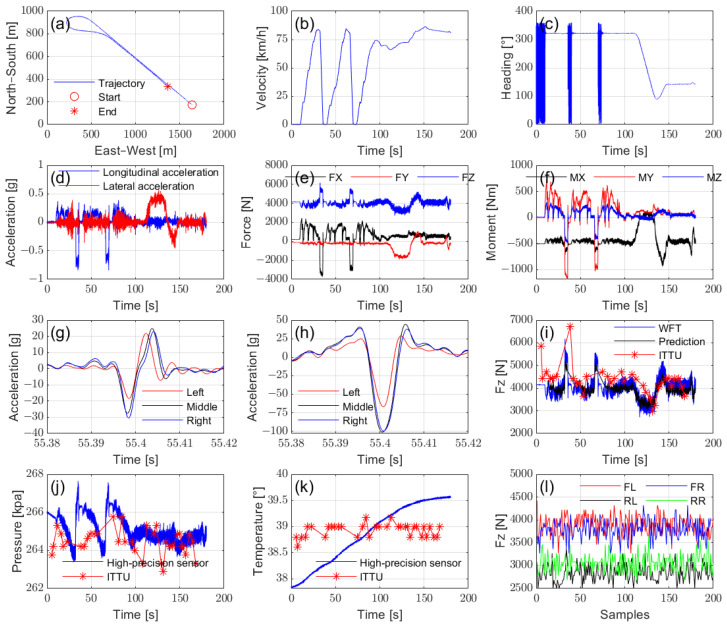
Road test data curves: (**a**) trajectory, (**b**) vehicle velocity, (**c**) heading angle, (**d**) vehicle acceleration, (**e**) wheel forces, (**f**) wheel moments, (**g**) *x*-axis acceleration at different installation positions, (**h**) *z*-axis acceleration at different installation positions, (**i**) vertical load, (**j**) tire pressure, (**k**) tire temperature, (**l**) vertical load measured by ITTU for all 4 tires during constant-speed straight-line driving.

Figure 20 presents key data from the road tests collected using the proposed test system. The road test consisted of two phases of acceleration and deceleration, followed by a turn and straight-line driving, as shown in the GPS trajectory, velocity, and heading data (Figure 20a–d). The vehicle speed ranged from 0 to nearly 90 km/h, covering typical driving conditions (Figure 20b). Additionally, vertical load shifts were observed in the multi-dimensional wheel force data (Figure 20e,f), while the tire pressure fluctuated with the driving conditions (Figure 20j), and the tire temperature increased accordingly (Figure 20k). During driving, in-tire accelerometers at different installation positions exhibited distinct characteristics, as illustrated in Figure 20g,h. All these parameters were successfully monitored by our system.

The integrated and synchronized data acquisition of the intelligent tire product ITTU (Figure 20i–l) enables the evaluation and calibration of a product, as well as algorithm fault tracing through a comparison with high-performance raw sensor data. Specifically, Figure 20j illustrates the effectiveness of vertical load estimation using intelligent tire technology by presenting the ground truth $F_{z}$ from the WFT, the estimated $F_{z}$ based on the data from the in-tire accelerometer, and the $F_{z}$ provided by the ITTU with the deployed estimation algorithm. Additionally, the data from all four tires (Figure 20l) can be utilized for further investigation into the integration of intelligent tire technology with vehicle dynamics, achieved through the combined analysis of vehicle motion data and wheel force data.

In summary, these data demonstrate the effectiveness of the proposed test system in supporting the validation and product-level assessment of intelligent tire technology in real-world road scenarios, with detailed data analysis to be explored in future studies.

## 6. Conclusions

### 6.1. Summary of Testing System Design and Functionality

This paper introduces a novel testing platform that supports the full lifecycle development of intelligent tire technologies, encompassing both bench testing and road testing. Extensive data collection is facilitated through advanced sensor integration, covering tire performance (in-tire accelerations, strains, pressure, temperature, and rotational dynamics), dynamic wheel forces (WFT), vehicle motion (GPS and inertial navigation), and driving behaviors (vehicle CAN bus). The system adopts a modular and distributed architecture, ensuring real-time performance at the lower layers (FPGA and RT) while providing advanced computational capabilities at the higher layers (PC). This design enables high-precision, multi-channel sensing with sampling rates of up to 50 kHz, offering a reliable and adaptable solution for both laboratory and real-world scenarios.

The system was validated in a tire vertical load estimation study, demonstrating the superior accuracy of accelerometers in vertical load estimation, confirming the effectiveness of the estimation algorithms and intelligent tire products, and highlighting the capability of the developed system to effectively support the advancement of intelligent tire technologies.

### 6.2. Potential for Future Research and Applications

The synchronized datasets from these experiments lay a foundation for further analysis and optimization. The PVDF sensor showed instability in predicting contact characteristics and the vertical load, highlighting the need for deeper investigation into raw sensor signals. The dynamic load data also support the future performance analysis of intelligent tire sensors.

These achievements underscore the system’s potential to drive innovation in intelligent tire research [46], enabling advanced studies of areas such as the following:

- **Road Validation and Practical Deployment:** The system’s adaptability to real-world testing facilitates the validation of laboratory results across diverse scenarios, bridging the gap between research and practical application.
- **The Exploration of Additional Tire Characteristics:** Leveraging a modular and extensible design, the system allows for the easy integration of additional sensors, enabling the exploration of new tire characteristics and sensor types.
- **The Optimization of Sensing Methods:** The multi-channel, multi-sensor setup facilitates the optimization of sensing configurations, sensor placement, and data fusion techniques across multiple sensor types, thereby improving the measurement accuracy and overall performance.
- **Integration with Vehicle Systems:** By synchronously capturing high-fidelity vehicle dynamics and driver behavior data, the system establishes a robust foundation for integrating intelligent tire sensing into vehicle control systems, thereby advancing applications such as ADAS and autonomous driving.

In conclusion, this study presents a robust testing system that addresses key challenges in intelligent tire development, offering significant potential for advancing both research and real-world applications.

## Figures and Tables

**Table 1:** Commonly studied parameters in intelligent tire technology that influence sensor outputs and serve as research targets.

Category	Parameter	Reference
Tire condition and performance	Pressure	[22]
	Temperature	[35]
	Wear	[22,24]
	Rotation speed	[3]
Tire–road interaction	Longitudinal force, $F_{x}$	[3,19]
	Lateral force, $F_{y}$	[3,11,19]
	Vertical force, $F_{z}$	[3,11,19,22,25]
	Aligning moment, $M_{z}$	[11]
	Contact patch length	[11,25]
	Slip ratio	[3]
	Slip angle	[3,11,25]
Vehicle motion and driving behaviors	Vehicle velocity	[3,22,25]
	Acceleration	[3,19]
	Braking	[3,19]
	Turning	[3,19]
Road condition	Friction coefficient	[11]
	Road surface classification	[5]

**Table 2:** MAPE of vertical load estimation using different methods.

Sensor	SVM (%)	Linear Regression (%)
Accelerometer	3.7271	8.7771
PVDF sensor	4.0686	19.1354

## Data Availability

Data are contained within the article.

## Abbreviations

The following abbreviations are used in this manuscript:

WFT	Wheel force transducer
PVDF	Polyvinylidene Fluoride
FPGA	Field-Programmable Gate Array
RT	Real-time controller
PC	Personal Computer
IEPE	Integrated Electronics Piezoelectric
TPMS	Tire Pressure Monitoring System
RTD	Resistance Temperature Detector
GPS	Global Positioning System
IMU	Inertial Measurement Unit
CAN	Controller Area Network
RTK	Real-Time Kinematic
TDMS	Technical Data Management Streaming
SVM	Support Vector Machine
RBF	Radial basis function
MAPE	Mean Absolute Percentage Error
ITTU	Intelligent Tire Test Unit
